# (6a*S*,11a*R*,11c*S*)-8-Sulfanylidene-2,3,5,6,6a,7,11,11a,11b,11c-decahydro-3a,7a-diaza-1*H*,4*H*-benzo[*de*]anthracen-3a-ium chloride hemihydrate

**DOI:** 10.1107/S160053681100972X

**Published:** 2011-06-18

**Authors:** Liang Wang, Chun-Mei Zhang, Jun-Xiang Guo, Qiu-Ye Wu, Hong-Gang Hu

**Affiliations:** a School of Pharmacy, Yantai University, Yantai 264005, People’s Republic of China; b Department of Organic Chemistry, School of Pharmacy, Second Military Medical University, Shanghai 200433, People’s Republic of China

## Abstract

The title compound, C_15_H_23_N_2_S^+^·Cl^−^·0.5H_2_O, was prepared from (6a*S*,11a*R*,11c*S*)-2,3,5,6,6a,7,11,11a,11b,11c-deca­hydro-3a,7a-diaza-1*H*,4*H*-benzo[*de*]anthracene-8-one (sophocarpine) and Lawesson’s reagent. The thione-substituted ring is in an envelope conformation and the three other six-membered rings are in chair conformations. In the crystal, anions and cations are linked by N—H⋯Cl and weak C—H⋯Cl hydrogen bonds. One 0.5-occupancy solvent water mol­ecule lies on a twofold rotation axis and another 0.25-occupancy solvent water mol­ecule is in a general position. The H atoms of these water mol­ecules were not located or included in the refinement.

## Related literature

For background to the medicinal uses of sophocarpine natural products, see: Gao *et al.* (2009 ▶); Jiang *et al.* (2007 ▶); Liu *et al.* (2007 ▶). For related structures, see: Ding *et al.* (2005 ▶); Khan *et al.* (1992 ▶). For the synthesis, see: Kaleta *et al.* (2006 ▶).
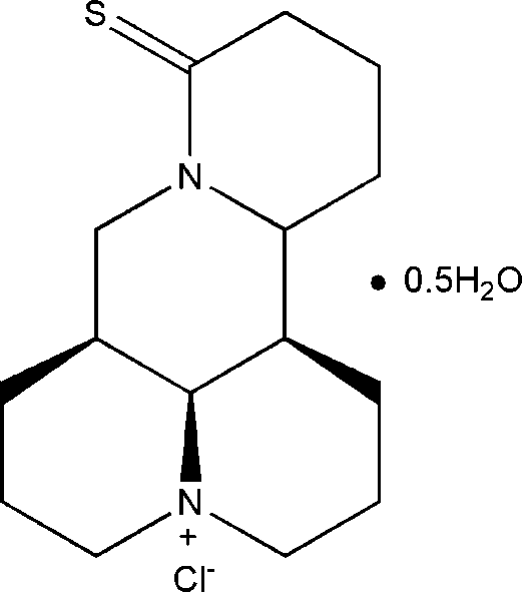


## Experimental

### 

#### Crystal data

C_15_H_23_N_2_S^+^·Cl^−^·0.5H_2_O
*M* *_r_* = 306.87
Tetragonal, 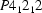

*a* = 7.793 (5) Å
*c* = 52.59 (5) Å
*V* = 3194 (4) Å^3^
*Z* = 8
Mo *K*α radiation
μ = 0.36 mm^−1^
*T* = 293 K
0.25 × 0.20 × 0.18 mm

#### Data collection

Bruker SMART CCD diffractometer
Absorption correction: multi-scan (*SADABS*; Sheldrick, 1996 ▶) *T*_min_ = 0.915, *T*_max_ = 0.937
11100 measured reflections
2816 independent reflections
2489 reflections with *I* > 2σ(*I*)
*R*_int_ = 0.043

#### Refinement

*R*[*F* ^2^ > 2σ(*F* ^2^)] = 0.071
*wR*(*F* ^2^) = 0.204
*S* = 1.23
2816 reflections
190 parameters
H atoms treated by a mixture of independent and constrained refinement
Δρ_max_ = 0.64 e Å^−3^
Δρ_min_ = −0.25 e Å^−3^
Absolute structure: Flack (1983 ▶), 1023 Friedel pairs
Flack parameter: 0.1 (2)

### 

Data collection: *SMART* (Bruker, 1997 ▶); cell refinement: *SAINT* (Bruker, 1997 ▶); data reduction: *SAINT*; program(s) used to solve structure: *SHELXS97* (Sheldrick, 2008 ▶); program(s) used to refine structure: *SHELXL97* (Sheldrick, 2008 ▶); molecular graphics: *SHELXTL* (Sheldrick, 2008 ▶); software used to prepare material for publication: *SHELXTL*.

## Supplementary Material

Crystal structure: contains datablock(s) global, I. DOI: 10.1107/S160053681100972X/lh5190sup1.cif

Structure factors: contains datablock(s) I. DOI: 10.1107/S160053681100972X/lh5190Isup2.hkl

Additional supplementary materials:  crystallographic information; 3D view; checkCIF report

## Figures and Tables

**Table 1:** Hydrogen-bond geometry (Å, °)

*D*—H⋯*A*	*D*—H	H⋯*A*	*D*⋯*A*	*D*—H⋯*A*
N1—H1⋯Cl1	1.10 (9)	2.01 (8)	3.019 (6)	151 (6)
C4—H4*A*⋯Cl1^i^	0.97	2.82	3.726 (7)	155

Symmetry code: (i) 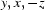.

**Fig. 1.**
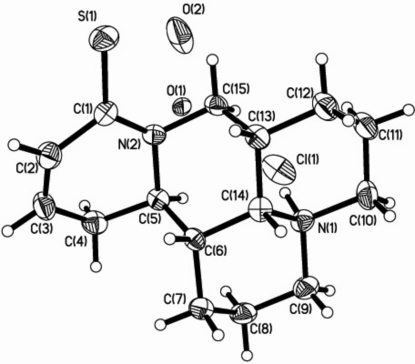
The molecular structure of the title compound with the atom-labeling scheme. Displacement ellipsoids are drawn at the 30% probability level.

## supplementary crystallographic information

### Comment

Sophocarpine ((6aS,11aR,11cS)-2,3,5,6,6a,7,11,11*a*,11*b*,11*c*- decahydro-1H,4H -3a,7a-diaza-βenzo[de] anthracene-8-one) and its derivatives have been found to possess a variety of pharmacological effects (Jiang *et al.*, 2007), including anti-inflammation (Liu *et al.*, 2007) and immunity-regulation activity (Gao *et al.* 2009). As part of our ongoing investigation of sophocarpine and its derivatives, we report here the crystal structure of the title compound.

The molecular structure of the title compound is shown in Fig. 1. The bond lengths and angles are comparable to related structures (Khan *et al.*, 1992; Ding *et al.*, 2005). The C1/C2/C3/C4/C5/N2 ring is in an envelope conformation with C5 forming the flap. The three other six-membered rings A(N1/C6/C7/C8/C9/C14), B(N1/C10/C11/C12/C13/C14) and C(N2/C1/C2/C3/C4/C5) are in chair conformations. In the crystal, anions and cations are linked by N—H···Cl and weak C—H···Cl hydrogen bonds.

### Experimental

The synthetic procedure followed the methods of Kaleta  *et al.* (2006). (6aS,11aR,11cS)-2,3,5,6,6a,7,11,11*a*,11*b*,11*c*-decahydro-1H, 4*H*-3a,7a-diaza-benzo[de] anthracene-8-one (0.01 mol) and Lawesson's reagent (0.04 mol) were mixed in toluene (100 ml) and stirred under reflux for 2 h. TLC showed that the reaction was completed. The mixture was concentrated and the residue was purified by a silica gel column. The products were stirred in dichloromethane and HCl was added. The resulting crystals were filtered and dried at room temperature. Single crystals suitable for X-ray measurements were obtained by recrystallization of a solution of the title compound in H_2_O at room temperature.

### Refinement

H atoms bonded to C atoms were fixed geometrically and allowed to ride on their attached atoms, with C—H distances in the range 0.93–0.98Å and with *U*_iso_(H) = 1.2*U*_eq_(C). The H atom bonded to N was refined independently with an isotropic displacement parameter. The H atoms of the partially occupied water molecules could not be located and are not included in the refinement although they are included in the formula.

### Crystal data

**Table:**

C_15_H_23_N_2_S^+^·Cl^−^·0.5H_2_O	*D*_x_ = 1.281 Mg m^−^^3^
*M**_r_* = 306.87	Mo *K*α radiation, λ = 0.71073 Å
Tetragonal, *P*4_1_2_1_2	Cell parameters from 927 reflections
Hall symbol: P 4abw 2nw	θ = 2.7–24.0°
*a* = 7.793 (5) Å	µ = 0.36 mm^−^^1^
*c* = 52.59 (5) Å	*T* = 293 K
*V* = 3194 (4) Å^3^	Block, yellow
*Z* = 8	0.25 × 0.20 × 0.18 mm
*F*(000) = 1320	

### Data collection

**Table:**

Bruker SMART CCD diffractometer	2816 independent reflections
Radiation source: fine-focus sealed tube	2489 reflections with *I* > 2σ(*I*)
graphite	*R*_int_ = 0.043
φ and ω scans	θ_max_ = 25.0°, θ_min_ = 2.6°
Absorption correction: multi-scan (*SADABS*; Sheldrick, 1996)	*h* = −9→7
*T*_min_ = 0.915, *T*_max_ = 0.937	*k* = −7→9
11100 measured reflections	*l* = −54→62

### Refinement

**Table:**

Refinement on *F*^2^	Secondary atom site location: difference Fourier map
Least-squares matrix: full	Hydrogen site location: inferred from neighbouring sites
*R*[*F*^2^ > 2σ(*F*^2^)] = 0.071	H atoms treated by a mixture of independent and constrained refinement
*wR*(*F*^2^) = 0.204	*w* = 1/[σ^2^(*F*_o_^2^) + (0.104*P*)^2^ + 2.0841*P*] where *P* = (*F*_o_^2^ + 2*F*_c_^2^)/3
*S* = 1.23	(Δ/σ)_max_ = 0.002
2816 reflections	Δρ_max_ = 0.64 e Å^−^^3^
190 parameters	Δρ_min_ = −0.25 e Å^−^^3^
0 restraints	Absolute structure: Flack (1983), 1023 Friedel pairs
Primary atom site location: structure-invariant direct methods	Flack parameter: 0.1 (2)

### Special details

**Table:**

Geometry. All e.s.d.'s (except the e.s.d. in the dihedral angle between two l.s. planes) are estimated using the full covariance matrix. The cell e.s.d.'s are taken into account individually in the estimation of e.s.d.'s in distances, angles and torsion angles; correlations between e.s.d.'s in cell parameters are only used when they are defined by crystal symmetry. An approximate (isotropic) treatment of cell e.s.d.'s is used for estimating e.s.d.'s involving l.s. planes.
Refinement. Refinement of *F*^2^ against ALL reflections. The weighted *R*-factor *wR* and goodness of fit *S* are based on *F*^2^, conventional *R*-factors *R* are based on *F*, with *F* set to zero for negative *F*^2^. The threshold expression of *F*^2^ > σ(*F*^2^) is used only for calculating *R*-factors(gt) *etc*. and is not relevant to the choice of reflections for refinement. *R*-factors based on *F*^2^ are statistically about twice as large as those based on *F*, and *R*- factors based on ALL data will be even larger.

### Fractional atomic coordinates and isotropic or equivalent isotropic displacement parameters (Å^2^)

**Table:**

	*x*	*y*	*z*	*U*_iso_*/*U*_eq_	Occ. (<1)
S1	0.6279 (2)	0.2217 (2)	0.11342 (2)	0.0559 (4)	
Cl1	0.2534 (2)	0.6515 (3)	0.01987 (3)	0.0785 (6)	
N1	0.4499 (5)	0.8604 (6)	0.05841 (7)	0.0415 (9)	
N2	0.6084 (5)	0.4228 (5)	0.07265 (6)	0.0357 (9)	
C1	0.7029 (6)	0.3215 (6)	0.08746 (8)	0.0400 (11)	
C2	0.8817 (7)	0.2948 (7)	0.08006 (9)	0.0511 (12)	
H2A	0.9548	0.2386	0.0913	0.061*	
C3	0.9422 (7)	0.3467 (8)	0.05836 (10)	0.0543 (13)	
H3A	1.0566	0.3260	0.0544	0.065*	
C4	0.8319 (7)	0.4387 (7)	0.03976 (9)	0.0483 (13)	
H4A	0.7864	0.3570	0.0276	0.058*	
H4B	0.9011	0.5211	0.0305	0.058*	
C5	0.6831 (6)	0.5324 (6)	0.05266 (7)	0.0354 (10)	
H5A	0.5944	0.5540	0.0398	0.043*	
C6	0.7298 (6)	0.7046 (6)	0.06520 (7)	0.0352 (10)	
H6A	0.8128	0.6796	0.0787	0.042*	
C7	0.8144 (7)	0.8338 (7)	0.04731 (10)	0.0482 (12)	
H7A	0.8599	0.9285	0.0572	0.058*	
H7B	0.9096	0.7789	0.0387	0.058*	
C8	0.6899 (7)	0.9026 (7)	0.02780 (10)	0.0537 (13)	
H8A	0.6499	0.8097	0.0170	0.064*	
H8B	0.7472	0.9864	0.0171	0.064*	
C9	0.5388 (7)	0.9856 (7)	0.04105 (10)	0.0557 (14)	
H9A	0.5785	1.0832	0.0509	0.067*	
H9B	0.4580	1.0271	0.0284	0.067*	
C10	0.2988 (7)	0.9449 (8)	0.07086 (11)	0.0562 (15)	
H10A	0.3382	1.0390	0.0814	0.067*	
H10B	0.2236	0.9916	0.0579	0.067*	
C11	0.2019 (7)	0.8205 (8)	0.08673 (10)	0.0558 (14)	
H11A	0.1539	0.7319	0.0759	0.067*	
H11B	0.1076	0.8793	0.0950	0.067*	
C12	0.3168 (7)	0.7373 (8)	0.10691 (10)	0.0562 (14)	
H12A	0.3504	0.8233	0.1193	0.067*	
H12B	0.2523	0.6490	0.1157	0.067*	
C13	0.4772 (6)	0.6578 (7)	0.09521 (8)	0.0422 (11)	
H13A	0.5549	0.6309	0.1093	0.051*	
C14	0.5717 (6)	0.7830 (6)	0.07802 (8)	0.0382 (11)	
H14A	0.6122	0.8773	0.0888	0.046*	
C15	0.4427 (6)	0.4900 (7)	0.08132 (8)	0.0394 (11)	
H15A	0.3878	0.4086	0.0926	0.047*	
H15B	0.3676	0.5098	0.0669	0.047*	
O1	0.2960 (17)	0.2869 (17)	0.0131 (3)	0.049 (3)	0.25
O2	0.0693 (14)	0.0693 (14)	0.0000	0.179 (12)	0.50
H1	0.390 (8)	0.755 (8)	0.0486 (10)	0.066 (17)*	

### Atomic displacement parameters (Å^2^)

**Table:**

	*U*^11^	*U*^22^	*U*^33^	*U*^12^	*U*^13^	*U*^23^
S1	0.0630 (9)	0.0610 (9)	0.0436 (6)	0.0009 (7)	−0.0016 (6)	0.0180 (6)
Cl1	0.0562 (9)	0.1312 (17)	0.0481 (7)	−0.0121 (10)	−0.0120 (7)	−0.0032 (8)
N1	0.044 (2)	0.041 (2)	0.0398 (19)	0.0012 (19)	−0.0050 (17)	0.0082 (18)
N2	0.037 (2)	0.040 (2)	0.0303 (17)	−0.0046 (17)	−0.0017 (15)	0.0024 (15)
C1	0.046 (3)	0.037 (3)	0.037 (2)	0.001 (2)	−0.006 (2)	−0.0013 (19)
C2	0.047 (3)	0.057 (3)	0.049 (3)	0.005 (3)	−0.007 (2)	0.007 (2)
C3	0.046 (3)	0.062 (4)	0.055 (3)	0.014 (3)	0.010 (2)	−0.001 (3)
C4	0.054 (3)	0.055 (3)	0.036 (2)	−0.002 (3)	0.008 (2)	−0.002 (2)
C5	0.034 (2)	0.046 (3)	0.0256 (19)	0.000 (2)	−0.0053 (17)	0.0039 (18)
C6	0.028 (2)	0.045 (3)	0.033 (2)	0.001 (2)	−0.0038 (17)	−0.0006 (19)
C7	0.041 (3)	0.048 (3)	0.056 (3)	−0.004 (2)	0.003 (2)	0.007 (2)
C8	0.056 (3)	0.052 (3)	0.052 (3)	−0.006 (3)	0.006 (2)	0.018 (2)
C9	0.056 (4)	0.048 (3)	0.064 (3)	0.002 (3)	0.000 (3)	0.021 (3)
C10	0.047 (3)	0.059 (3)	0.062 (3)	0.020 (3)	0.002 (3)	0.001 (3)
C11	0.044 (3)	0.074 (4)	0.049 (3)	0.013 (3)	0.011 (2)	0.008 (3)
C12	0.049 (3)	0.072 (4)	0.047 (3)	0.011 (3)	0.009 (2)	0.008 (3)
C13	0.038 (3)	0.056 (3)	0.032 (2)	0.003 (2)	−0.0029 (18)	0.007 (2)
C14	0.042 (3)	0.045 (3)	0.0281 (19)	0.000 (2)	−0.0086 (18)	−0.0039 (19)
C15	0.029 (2)	0.049 (3)	0.040 (2)	−0.001 (2)	−0.0022 (19)	0.012 (2)
O1	0.033 (7)	0.026 (7)	0.089 (9)	−0.006 (6)	0.007 (7)	0.002 (6)
O2	0.074 (7)	0.074 (7)	0.39 (3)	−0.027 (9)	0.080 (13)	−0.080 (13)

### Geometric parameters (Å, °)

**Table:**

S1—C1	1.676 (5)	C7—H7B	0.9700
N1—C10	1.499 (7)	C8—C9	1.513 (8)
N1—C9	1.505 (6)	C8—H8A	0.9700
N1—C14	1.526 (6)	C8—H8B	0.9700
N1—H1	1.07 (6)	C9—H9A	0.9700
N2—C1	1.331 (6)	C9—H9B	0.9700
N2—C15	1.466 (6)	C10—C11	1.485 (8)
N2—C5	1.474 (5)	C10—H10A	0.9700
C1—C2	1.462 (8)	C10—H10B	0.9700
C2—C3	1.299 (7)	C11—C12	1.532 (7)
C2—H2A	0.9300	C11—H11A	0.9700
C3—C4	1.486 (8)	C11—H11B	0.9700
C3—H3A	0.9300	C12—C13	1.525 (7)
C4—C5	1.529 (7)	C12—H12A	0.9700
C4—H4A	0.9700	C12—H12B	0.9700
C4—H4B	0.9700	C13—C14	1.520 (6)
C5—C6	1.539 (6)	C13—C15	1.522 (7)
C5—H5A	0.9800	C13—H13A	0.9800
C6—C7	1.527 (6)	C14—H14A	0.9800
C6—C14	1.531 (6)	C15—H15A	0.9700
C6—H6A	0.9800	C15—H15B	0.9700
C7—C8	1.511 (7)	O1—O1^i^	1.38 (3)
C7—H7A	0.9700		
			
C10—N1—C9	110.0 (4)	C7—C8—H8B	109.7
C10—N1—C14	111.5 (4)	C9—C8—H8B	109.7
C9—N1—C14	112.3 (4)	H8A—C8—H8B	108.2
C10—N1—H1	102 (3)	N1—C9—C8	111.1 (4)
C9—N1—H1	114 (3)	N1—C9—H9A	109.4
C14—N1—H1	107 (3)	C8—C9—H9A	109.4
C1—N2—C15	121.2 (4)	N1—C9—H9B	109.4
C1—N2—C5	122.8 (4)	C8—C9—H9B	109.4
C15—N2—C5	111.2 (4)	H9A—C9—H9B	108.0
N2—C1—C2	117.2 (4)	C11—C10—N1	111.0 (4)
N2—C1—S1	123.9 (4)	C11—C10—H10A	109.4
C2—C1—S1	118.9 (4)	N1—C10—H10A	109.4
C3—C2—C1	122.3 (5)	C11—C10—H10B	109.4
C3—C2—H2A	118.8	N1—C10—H10B	109.4
C1—C2—H2A	118.8	H10A—C10—H10B	108.0
C2—C3—C4	121.2 (5)	C10—C11—C12	111.6 (5)
C2—C3—H3A	119.4	C10—C11—H11A	109.3
C4—C3—H3A	119.4	C12—C11—H11A	109.3
C3—C4—C5	112.1 (4)	C10—C11—H11B	109.3
C3—C4—H4A	109.2	C12—C11—H11B	109.3
C5—C4—H4A	109.2	H11A—C11—H11B	108.0
C3—C4—H4B	109.2	C13—C12—C11	111.8 (4)
C5—C4—H4B	109.2	C13—C12—H12A	109.3
H4A—C4—H4B	107.9	C11—C12—H12A	109.3
N2—C5—C4	109.8 (4)	C13—C12—H12B	109.3
N2—C5—C6	107.0 (3)	C11—C12—H12B	109.3
C4—C5—C6	115.3 (4)	H12A—C12—H12B	107.9
N2—C5—H5A	108.2	C14—C13—C15	110.6 (3)
C4—C5—H5A	108.2	C14—C13—C12	112.1 (4)
C6—C5—H5A	108.2	C15—C13—C12	113.5 (4)
C7—C6—C14	110.8 (4)	C14—C13—H13A	106.7
C7—C6—C5	114.4 (4)	C15—C13—H13A	106.7
C14—C6—C5	110.3 (4)	C12—C13—H13A	106.7
C7—C6—H6A	107.0	C13—C14—N1	110.7 (4)
C14—C6—H6A	107.0	C13—C14—C6	113.3 (4)
C5—C6—H6A	107.0	N1—C14—C6	111.1 (3)
C8—C7—C6	112.0 (4)	C13—C14—H14A	107.1
C8—C7—H7A	109.2	N1—C14—H14A	107.1
C6—C7—H7A	109.2	C6—C14—H14A	107.1
C8—C7—H7B	109.2	N2—C15—C13	107.5 (4)
C6—C7—H7B	109.2	N2—C15—H15A	110.2
H7A—C7—H7B	107.9	C13—C15—H15A	110.2
C7—C8—C9	109.8 (4)	N2—C15—H15B	110.2
C7—C8—H8A	109.7	C13—C15—H15B	110.2
C9—C8—H8A	109.7	H15A—C15—H15B	108.5

Symmetry codes: (i) *y*, *x*, −*z*.

### Hydrogen-bond geometry (Å, °)

**Table:**

*D*—H···*A*	*D*—H	H···*A*	*D*···*A*	*D*—H···*A*
N1—H1···Cl1	1.10 (9)	2.01 (8)	3.019 (6)	151 (6)
C4—H4A···Cl1^i^	0.97	2.82	3.726 (7)	155

Symmetry codes: (i) *y*, *x*, −*z*.
